# Hand Hygiene Knowledge, Perception, and Practices among Domestic Visitors to the Prophet’s Mosque in Al Madinah City Amid the COVID-19 Pandemic: A Cross-Sectional Study

**DOI:** 10.3390/ijerph18020673

**Published:** 2021-01-14

**Authors:** Hashim A. Mahdi, Hamza M. Assaggaf, Mohammad Alfelali, Omar B. Ahmed, Radi Alsafi, Ramon Z. Shaban, Robert Booy, Harunor Rashid

**Affiliations:** 1 National Centre for Immunisation Research and Surveillance, The Children’s Hospital at Westmead, Westmead, NSW 2145, Australia; robert.booy@health.nsw.gov.au (R.B.); harunor.rashid@health.nsw.gov.au (H.R.); 2 Faculty of Medicine and Health, The University of Sydney, Sydney, NSW 2006, Australia; 3 Department of Public Health, College of Health Sciences, Saudi Electronic University, Jeddah 23442, Saudi Arabia; 4 Department of Environmental and Health Research, The Custodian of the Two Holy Mosques Institute for Hajj and Umrah Research, Umm Al Qura University, Makkah 24381, Saudi Arabia; hmsaggaf@uqu.edu.sa (H.M.A.); abuaglah1@hotmail.com (O.B.A.); rtsafi@uqu.edu.sa (R.A.); 5 The Custodian of the Two Holy Mosques Institute for Hajj and Umrah Research Al-Madinah Branch, Umm Al Qura University, Al-Madinah 42372, Saudi Arabia; 6 Department of Laboratory Medicine, Faculty of Applied Medical Sciences, Umm Al-Qura University, Makkah 24381, Saudi Arabia; 7 Department of Family and Community Medicine, Faculty of Medicine in Rabigh, King Abdulaziz University, Jeddah 22252, Saudi Arabia; malfelali@kau.edu.sa; 8 Faculty of Medicine and Health, Susan Wakil School of Nursing and Midwifery & Marie Bashir Institute for Infectious Diseases and Biosecurity, The University of Sydney, Sydney, NSW 2006, Australia; ramon.shaban@sydney.edu.au; 9 New South Wales Biocontainment Centre and the Department of Infection Prevention and Control, Division of Infectious Diseases and Sexual Health, Westmead Hospital and Western Sydney Local Health District, Westmead, NSW 2151, Australia; 10 Marie Bashir Institute for Infectious Diseases and Biosecurity, School of Biological Sciences and Sydney Medical School, The University of Sydney, Sydney, NSW 2145, Australia

**Keywords:** COVID-19, hand hygiene, knowledge, practice, visitors to the Prophet’s Mosque

## Abstract

This study aimed to assess hand hygiene knowledge, perception, and practices of visitors to the Prophet’s Mosque in Al Madinah City, Saudi Arabia. Using a self-administered electronic questionnaire, a cross-sectional survey was conducted among domestic residents, who visited the mosque between 31 July and 3 August 2020. Participants’ demographic data, hand hygiene knowledge, perception, and practices were collected. Four hundred participants aged 18–65 (median 36) years completed the survey, of which 215 (53.8%) were female. The visitors’ mean knowledge score about hand hygiene was 6.4 (± standard deviation (SD) 1.35) of total 12. Most participants (392, 98%) were aware of the role of hand hygiene in preventing Coronavirus Disease 2019 (COVID-19); nevertheless, 384 (96%) said hand hygiene lowers body immunity and 316 (79%) thought <60% alcohol is sufficient for hand disinfection. Males had a higher knowledge score than females (6.46 (±1.41) vs. 6.14 (±1.27), *p* = 0.02) and, visitors who had no formal education scored higher than those with post-graduate education (6.88 (±1.45) vs 5.73 (±1.12), *p* = 0.01). Washing hands with soap and water was the predominant method practiced after a meal (365, 91.7%), after toilet visit (354, 88.5%), after touching a surface (262, 65.7%), after waste disposal (332, 83.2%), and when hands were visibly dirty (357, 89.5%). Al Madinah visitors had moderate knowledge about hand hygiene, but demonstrated some knowledge gaps and negligence in practice that are crucial to curb the spread of COVID-19.

## 1. Introduction

A mass gathering (MG) is defined as a congregation of attendees at a specific location for a specific purpose over a period of time, and that has the potential to strain the planning and response resources of the host country or community [1]. Millions of Muslims from around the world travel every year to the Two Holy Mosques in Kingdom of Saudi Arabia (KSA): The Grand Mosque in the city of Makkah for Hajj and Umrah, and the Prophet’s Mosque (Al-Masjid al-Nabawi) in the city of Al Madinah that houses the shrine of the Prophet Mohammad (peace be upon him). This visitation to the Prophet’s Mosque, called ‘Ziyarah’ in Arabic, is not an integral part of Hajj, but is considered by pilgrims to be a unique revered optional opportunity that should not be missed. Hence, most Hajj and Umrah pilgrims’ itineraries include this Ziyarah [2], and they stay in Al Madinah for several days.

The global pandemic of the novel Coronavirus Disease 2019 (COVID-19) has affected more than 89 million individuals with fatalities exceeding 1.9 million around the world, and over 363,000 confirmed cases with over 6000 deaths in Saudi Arabia (as of 9 January 2021) [3]. Religious MGs pose a significant risk for the spread of respiratory tracts infections, as is currently the case with the COVID-19 pandemic [4,5]. Multiple outbreaks of COVID-19 in different countries have been traced to attending such religious events [6,7,8]. In order to prevent the spread of the virus among attendees of Hajj and Umrah MGs, several sectors in KSA have taken immediate and drastic measures from the beginning of this pandemic; for example, the Saudi Arabian authorities immediately imposed a lockdown in these holy sites and suspended Umrah and Ziyarah [9]. Since early June 2020, restrictions on the Prophet’s Mosque were eased gradually and a limited number of current residents of Saudi Arabia (domestic and expatriates) were permitted to pray with a strict public health protocol to follow, including observing hand hygiene [10].

Frequently and thoroughly washing hands with soap and water for at least 20 s or sterilising hands with a 60% alcohol-based hand sanitiser, when soap and water are not available, can help prevent respiratory viral infections in settings where people are more likely to mix, contract, and spread infections [11]. As COVID-19 can spread through contact with contaminated surfaces, hand hygiene remains a fundamental control and prevention measure and is strongly recommended to curb its transmission, especially in the absence of a clinically approved vaccine or antiviral prophylaxis [11].

Studies found that hand hygiene was the most favoured infection prevention method among Hajj pilgrims prior to the COVID-19 crisis [12,13,14]. A survey conducted by our team during the Hajj 2019 demonstrated that domestic Hajj pilgrims had sound hand hygiene knowledge and practice, but there are gaps in areas that are now considered vital to contain and mitigate outbreaks such as the COVID-19 pandemic [15]; however, anecdotally, it is believed that COVID-19 campaigns may have improved the situation. The Saudi general population have shown acceptable awareness and practices towards COVID-19 [16,17,18,19]. Nonetheless, there is no focused published study that has examined the knowledge and practice of hand hygiene among visitors to the Prophet’s Mosque in Al Madinah City during the pandemic or earlier. Therefore, this study aims to assess hand hygiene knowledge, perception, and practice among visitors to the Prophet’s Mosque amidst the COVID-19 pandemic.

## 2. Materials and Methods

Using a convenient sampling strategy, a cross-sectional survey was conducted among Saudi Arabian residents who visited the Prophet’s Mosque in Madinah, Saudi Arabia, during Eid al-Adha (the feast of sacrifice), corresponding to the last days of Hajj over four days starting from 31 July to 3 August 2020. Visitors of both genders aged ≥18 years were invited to participate, while the minors (i.e., persons under 18 years of age) and individuals not having a full mental capacity to comprehend the consent form in Arabic or English were excluded.

For the health and safety of the research team members and study participants amidst the ongoing outbreak of COVID19, a self-administered electronic questionnaire in Arabic or English was deemed preferable to a pen-paper based survey. Four trained data collectors approached the potential participants at the mosque and explained the study purpose and methodology. Visitors who agreed to participate were then given a tablet device where the questionnaire was completed, and the responses were recorded through an online survey development cloud-based software (Rased) (The Custodian of the Two Holy Mosques Institute for Hajj and Umrah Research, Makkah, Saudi Arabia). This being an anonymous survey, no signed consent was obtained, and respondents’ completion of the survey was considered as their implied consent. Ethics approval was obtained from King Abdullah Medical City (KAMC), Makkah, Saudi Arabia (IRB reference number: 20-661).

The questionnaire was devised using some exemplary questions used in a published survey conducted in 2019 by the study authors [15] and was revised by taking the COVID-19 pandemic into consideration and feedback from MG and infectious disease experts. Following the revision, the final version was prepared and grammatical discrepancies in both Arabic and English were fixed. The questionnaire consisted of three major parts. Part 1 collected non-identifiable socio-demographic information about the participant including gender, age, education, and nationality. Part 2 collected data about the participants’ knowledge of hand hygiene using true/false statements about risks, practice, and common myths and fallacies associated with hand hygiene reported in the literature and global public health institutes (the World Health Organization and the U.S. Centers for Disease Control and Prevention) [20,21]. The sum of knowledge scores was calculated for each participant using a scale from 0 to 12, with a higher score indicating considerable knowledge level on hand hygiene and a lower score indicating poor knowledge. Additionally, participants’ perception regarding the germicidal effects of different hand hygiene methods including against severe acute respiratory syndrome coronavirus 2 (SARS-CoV-2) was also captured in Section 2. Part 3 comprised items related to self-reported hand hygiene behaviour. Participants were asked how often they used different hand hygiene products (water only, soap and water, and alcohol-based hand rubs (ABHRs)) to clean their hands per day in the past two weeks prior to data collection. They were also asked which of these products they used for cleaning hands during key situations when they are most likely to be exposed to germs such as before and after eating, after toileting, and so on. Finally, participants were asked to report what possible reasons may have hindered them from practising regular hand hygiene and what side effects they experienced from using hand hygiene products.

All the collected data in the software were exported to a master Excel spreadsheet (Microsoft Office 356, version 2002, Redmond, WA, USA) for cleaning and coding before importing to Statistical Package for Social Sciences (SPSS) software (IBM SPSS Statistics for Windows, version 26.0, IBM Corp, Armonk, NY, USA). Frequencies and percentages were used to present the categorical variables including most demographic data, hand hygiene knowledge level, perception, and practices, while the mean (or median with range) ± standard deviation (SD) was used to summarise continuous variables including total hand hygiene knowledge score. Pearson correlation test was used to indicate the strength of association between visitors’ age and their level of hand hygiene knowledge, while one-way analysis of variance (ANOVA) was applied to determine the significant differences of the sample categorical characteristics in association with the knowledge score. Pearson’s chi-squared test was applied to assess differences among the sample characteristics and participants’ practice of hand hygiene. A *p*-value ≤ 0.05 was considered to be statistically significant.

## 3. Results

Of total 412 individuals approached, all (100%) agreed to take part in the study, of whom 400 (97%) participants aged 18–65 (median of 36) years completed the online questionnaire, of whom 215 (53.8%) were female. The local Saudi citizens accounted for 83 (21.7%) of the sample, with the leading expatriates being Pakistanis 84 (22%); further, 284 (71.7%) resided in Madinah, 146 (36.7%) had a high school level education, and 52 (13%) reported having at least one comorbidity (Table 1).

The findings of the participants’ knowledge level of hand hygiene are detailed in Table 2. The mean (±SD) hand hygiene knowledge score was 6.4 (±1.35), a quarter (98, 24.5%) of the participants had a poor knowledge score of <6, nearly three-quarters (297, 74.3%) had a medium score of 6 to 9, and a small proportion (5, 1.3%) had a high score of ≥10. Men had a significantly higher hand hygiene knowledge mean score than women (6.46 (±1.41) vs 6.14 (±1.27), *p* = 0.02); however, participants with no formal education had a higher hand hygiene knowledge score than those with post-graduate level education (6.88 (±1.45) vs 5.73 (±1.12), *p* = 0.01). Fewer participants correctly responded to the following knowledge statements: HIV/AIDS does not transmit through poor hand hygiene (34.5%), keeping hands clean does not effect on immunity system (4%), ABHRs should contain at least 60% of alcohol for disinfection of hands (21%), antiseptic/antibacterial soaps are not more effective at preventing infections than washing with plain soap (2.5%), and water temperature does not make a difference in the cleansing effect of washing of hands (45.8%).

Table 3 shows that most participants believed cleansing hands with antiseptic/antibacterial soap and water (263, 65.8%), plain soap and water (195, 48.8%), and alcohol-based hand rubs (224, 56%) were very effective hand hygiene methods, and 60 (15%) thought even plain water is very effective.

Table 4 presents the frequency of hand hygiene practice methods across both genders, showing that males used soap and water more frequently than females (*p* = 0.05). Table 5 summarises participants’ hand hygiene practices and methods used before and after nine key actions showing water alone or soap and water was the predominant hand hygiene method practised before a meal, by 215 (53.9%) and 165 (41.4%) participants, respectively, while soap and water was the main method used after a meal (365, 91.7%), after toilet visit (354, 88.5%), after touching a surface (262, 65.7%), after caring for a patient (213, 53.4%), when hands were visibly dirty (357, 89.5%), after waste disposal (332, 83.2%), and after handshakes (112, 28%). More people used disposable handkerchiefs (110, 27.6%) and soap and water (102, 25.6%) following a sneeze or cough.

The main reported reasons that might have hindered the visitors from complying with regular hand hygiene were failure to remember (210, 52.5%), being too busy (96, 24%), and difficulty in accessing hand cleaning products (91, 22.8%). A small number (92, 23.1%) of participants experienced adverse dermatologic effects of adopting frequent hand hygiene, such as skin dryness, peeling skin, allergy, or eczema, which of whom 64 (70%) were female.

## 4. Discussion

To the best of our knowledge, this is the first study that focused on evaluating knowledge, perception, and reported practices of hand hygiene among domestic visitors to the Prophet’s mosque in Al Madinah City amidst the COVID-19 pandemic. As elaborated below, the key findings of this survey may inform future policy.

### 4.1. Knowledge about Hand Hygiene

The participants in this study achieved a moderate level of the knowledge about hand hygiene, with an average score of 6.4 (±1.35), and nearly three-quarters (297, 74.25%) obtained a medium score. This finding indicates that most study participants (392, 98%) were knowledgeable about the protective role of a good hand hygiene practice against the COVID-19 and other respiratory infections, yet only one-third correctly reported that HIV/AIDS does not transmit through poor hand hygiene. According to the visitors’ responses, poor knowledge was detected in numerous aspects associated with the common myths surrounding hand hygiene and a proper hand hygiene procedure. For example, 96% of participants mistakenly reported that consistent hand washing negatively impacts body immunity, almost 80% were unaware of the correct concentration of alcohol hand washing solutions should contain to ensure optimum disinfection, and more than half thought the temperature of the water makes a difference in terms of the cleansing effect of hand washing. Concerningly, many visitors were unaware that engaging in high-risk practices, such as applying bleach or other household disinfectant products to bare hands and using ultra-violet lamps to disinfect hands, can damage skin tissues [20,22]. Anticipating such high-risk practices with the intention to combat COVID-19 was observed in a survey conducted among U.S. residents [22].

Despite the intensive awareness-raising COVID-19 campaigns, the hand hygiene knowledge score in this survey did not significantly improve—rather, it slightly worsened—compared with our previous survey conducted among Hajj pilgrims in 2019 (average knowledge score in this survey 6.4 compared with 6.7 in the previous year) [15]. Nonetheless, some statements in the current study received higher correct responses like the essential role of cleaning of hands in preventing respiratory illnesses transmission (98% versus 78%) and the accurate time (at least 20 s) of scrubbing hands with soap (94% versus 42%). These higher rates of correct responses probably indicate that people have become more aware of the protective role of hand hygiene and its correct practices amidst the ongoing COVID-19 pandemic. This fact is supported by several recent surveys that determined the awareness level and practice toward COVID-19 among the Saudi Arabian population. For instance, an online survey involving 6000 members of Saudi public showed 22.4% of respondents indicated that washing hands with soap and water was important to avoid transmission of COVID-19 [16]; in another survey of 3388, 87.7% strongly agreed and 10.8% just agreed that hand hygiene was essential to protect oneself from COVID-19; and in a third survey involving 443 respondents [17], 96% of respondents reported they knew they had to wash their hands with soap and water for 20 s, however, 16.2% were not compliant with this knowledge [18].

Gender and education level were identified to be predictive factors in this study. Male participants tended to have a higher level of hand hygiene knowledge and were more likely to wash their hands with soap and water more frequently (>5 times/day). This could be because males are mostly outdoor, and hence are more exposed to healthcare messages, or maybe because males are more careful about their health as COVID-19 is worse in males [23]. Surprisingly, education level was negatively associated with knowledge, where participants who did not attain any academic certificate were more likely to score higher in the knowledge level than postgraduate degree holders. On the contrary, some recent findings among the Saudi population suggested that females and highly educated people are more adherent to and knowledgeable about COVID-19 prevention and control measures [17,19]. This could be a chance finding, and hence needs further investigation. This also hints that perhaps more focused health education is needed to improve public health, irrespective of background educational status of a person.

### 4.2. Perception of Hand Hygiene

Most of the visitors to the mosque had a positive perception of the effectiveness of hand hygiene in killing viruses existing in our hands, including COVID-19, and this matched the results of studies involving the Saudi general population and healthcare workers, which found that over 90% agreed that washing hands is essential to protect themselves and others from COVID-19 transmission [17,24]. Noticeably, participants had a strong belief that antiseptic/antibacterial soaps are more effective than plain soap in preventing infectious illnesses, while evidence to date has not proven that there is a difference between the use of these products on the effectiveness of hand cleansing. In addition, long-term exposure to antibacterial substances in hand hygiene products may increase the risk of developing antimicrobial resistance [25].

### 4.3. Practice of Hand Hygiene

When participants were asked about their hand hygiene practices at specific high-risk situations, soap and water was the most preferred method reportedly used, followed by alcohol-based hand rubs. This finding is in parallel with the results of formerly conducted studies among attendees of Hajj and Umrah pilgrimage that found hand washing with soap and water was more prevalent than other hand hygiene methods [15,26,27,28]. It is also noteworthy that Saudi Arabian citizens have a preference for using soap and water to ABHR as a preventive measure against COVID-19 [19]. In spite of the recommendations of washing hands after sneezing or coughing and following handshakes to prevent contracting and spreading infections, it seems that the low compliance of hand hygiene during these situations is an issue that persists even during the pandemic. This survey shows a relatively poor hand hygiene behaviour after sneezing or coughing, with only 25.6% washing hands with soap and water and 12.6% using alcoholic hand sanitiser, as well as following handshakes (28% and 26.8%, respectively), and the largest proportion (27.6%) used a handkerchief following a sneeze, while previously, they washed hands with water and soap [15]. Similarly, approximately 27% of the Saudi Arabian citizens did not wash their hands after nose-blowing, coughing, or sneezing amidst the COVID-19 pandemic [17]. Conversely, hand hygiene compliance in this study improved slightly following other high-risk actions, e.g., about 98% of visitors of the mosque cleaned their hands after touching a patient and 99% cleaned hands following waste disposal, whereas 85% and 90% of pilgrims, respectively, during the 2019 Hajj complied [15]. Another survey involving Umrah pilgrims conducted in 2019 showed that over 90% of pilgrims washed their hands with soap and water or sanitisers after coughing and sneezing, before eating or preparing food, and after using the bathroom [29]. The fact that fewer participants were complying with ABHR compared with using soap and water may have stemmed from the religious and cultural preference of Muslims to avoid alcoholic substances when equivalent alternates are available [30].

### 4.4. Barriers and Side Effects of Hand Hygiene

Visitors reported that being busy, forgetting to clean hands when they should, and inaccessibility sometimes to hygienic products were the main obstacles, among others, to regular hand hygiene. Hence, people intending to participate in MG events are encouraged to carry their personal hygiene products to overcome these problems and increase the uptake. Few visitors (92, 23.1%), mostly women, reported having abnormal skin reactions as a result of frequent hands washing. Some advisable tips, such as applying moisturizers after hand cleaning, are helpful in preventing further side effects [31].

### 4.5. Implications for the Upcoming Religious Mass Gatherings

The Saudi Arabian health authorities have launched a nationwide COVID-19 awareness campaign using various sources (e.g., social media platforms) to disseminate information about the disease rout of transmission, signs and symptoms, and preventive strategies; in turn, this effort, along with other drastic measures, has helped significantly in reducing the newly acquired cases and controlling the spread of the disease in the country [32]. More people have been attending the Two Holy Mosques, since October 2020, following the Saudi government decision to ease more restrictions on visiting of the Prophet’s mosque, and gradually reopening Umrah season to pilgrims in Makkah after a lockdown that lasted for seven months [10]. Therefore, COVID-19 precautionary messages should continue to target this population with an emphasis on evidence-based instructions about the best and safe hygiene practices.

### 4.6. Study Limitations and Future Recommendations

The major limitation of this study was that it only captured the knowledge and practices of visitors to the Prophet’s mosque in a certain stage of the pandemic (between 31 July and 3 August 2020); consequently, the results may not be generalizable outside the domain of the defined population and scope of the current study. We recommend implementing an additional survey involving participants of another religious MG setting in Saudi Arabia (such as Umrah) to assess hand hygiene awareness and practices at a different stage of the pandemic.

## 5. Conclusions

There is a lack of research investigating hand hygiene at religious mass events during the COVID-19 pandemic. This study showed that the domestic visitors to the Prophet’s mosque had a moderate knowledge score of hand hygiene with consideration of variable knowledge gaps, specifically in some aspects related to accurate hand hygiene techniques and involvement in high-risk practices. The participants reported better practices of hand hygiene during the current COVID-19 disaster, yet negligence in the compliance has been observed in some situations that could lead to contracting and disseminating infections. Sustained improvements through intensive awareness-raising interventions to enhance knowledge and practice of hand hygiene are essential for MG participants from different sociodemographic backgrounds amidst the ongoing COVID-19 pandemic.

## Figures and Tables

**Table 1:** Demographic characteristics of respondents (*n* = 400).

Characteristics	Findings
Median (range) of age in years	36 (18–65)
Male: Female *n* (%)	185: 215 (46.3: 53.8)
Nationality *n* (%)	
Saudi Arabia	83 (21.7)
Pakistan	84 (21.9)
Afghanistan	23 (6)
Bangladesh	19 (5)
Egypt	28 (7.3)
India	35 (9.1)
Sudan	23 (6)
Yemen	19 (5)
Others	69 (18)
Residence in Saudi Arabia *n* (%)	
Al Madinah	284 (71.7)
Jeddah	43 (10.9)
Yanbu	9 (2.3)
Riyadh	31 (7.8)
Al-Qassim	6 (1.5)
Hail	6 (1.5)
Others	17 (4.3)
Education *n* (%)	
None	26 (6.5)
Primary/elementary school certificate	68 (17.1)
High school certificate	146 (36.7)
Diploma	43 (10.8)
University degree	78 (19.6)
Higher university degree	37 (9.3)
Presence of pre-existing medical conditions *n* (%)	
Absent	348 (87)
Present	
Hypertension	35 (8.8)
Chronic heart diseases	6 (1.5)
Diabetes	17 (4.3)
Chronic kidney disease	4 (1)
Chronic gastrointestinal disease	8 (2)

**Table 2:** Hand hygiene knowledge level among participants (*n* = 400).

Statements	Correct Answer *n* (%)
1. Respiratory infections including “COVID-19” can be transmitted by poor hand hygiene	392 (98)
2. HIV/AIDS can be transmitted by poor hand hygiene	138 (34.5)
3. Always keeping your hands clean may lower our body defence mechanism	16 (4)
4. ABHR containing less than 60% of alcohol is sufficient for hands disinfection	84 (21)
5. Washing of and with soap and water from 20 to 40 s is sufficient for hands disinfection	376 (94)
6. Hands should be held under water while lathering with soap	249 (62.3)
7. The temperature of water does not make difference in terms of the cleansing effect of hand washing	183 (45.8)
8. Antiseptic/antibacterial soaps are more effective at preventing illness than washing with plain soap and water	10 (2.5)
9. Using ABHR may not be as effective as the use of soap and water when hands are visibly dirty	359 (89.8)
10. There is no need to clean our hands after sneezing, coughing, or shaking hands	291 (72.8)
11. Ultra-violet (UV) lamps should not be used to disinfect our hands or other areas of our skin	187 (46.8)
12. Spraying and introducing bleach or another surface disinfectant into our hands is safe and it will protect us against COVID-19 and other infections	231 (57.8)
Mean (±SD) of total scores (0 to 12)	6.4 (±1.35)

ABHR = alcohol-based hand rub; COVID-19 = Coronavirus Disease 2019.

**Table 3:** Perceptions of the effectiveness of hand hygiene methods (*n* = 400).

Participants’ Perception of Effectiveness	*n* (%)
Water only	
Not effective	67 (17)
Somewhat effective	272 (68)
Very effective	60 (15)
Plain soap and water	
Not effective	3 (0.8)
Somewhat effective	202 (50.5)
Very effective	195 (48.8)
Antiseptic/antibacterial soap and water	
Not effective	1 (0.3)
Somewhat effective	136 (34)
Very effective	263 (65.8)
ABHR	
Not effective	2 (0.5)
Somewhat effective	174 (43.5)
Very effective	224 (56)

ABHR = alcohol-based hand rub.

**Table 4:** Frequency of reported practice of hand hygiene methods in both gender groups (*n* = 400).

Hand Hygiene Practices by Gender Groups	Never *n* (%)	Less Frequently (≤5 Times/Day) *n* (%)	More Frequently (>5 Times/Day) *n* (%)	*p*-Value ^a^
Water only				
Male (*n* = 185)	4 (2)	52 (28)	129 (70)	0.08
Female (*n* = 214)	1 (0.5)	78 (36.5)	135 (63)	
Soap and water				
Male (*n* = 185)	1 (0.5)	146 (79)	38 (20.5)	0.05 *
Female (*n* = 215)	1 (0.5)	189 (88)	25 (11.5)	
ABHR				
Male (*n* = 184)	11 (6)	170 (92.4)	3 (1.6)	0.30
Female (*n* = 215)	22 (10)	190 (88.4)	3 (1.6)	

^a^ By chi-square test; asterisks * indicate a significant difference; ABHR = alcohol-based hand rub.

**Table 5:** Participants’ hand hygiene practices and methods used in the nine key actions (*n* = 400).

Types of Hand Hygiene	Before Meal *n* (%)	After Meal *n* (%)	After Toileting *n* (%)	After Touching a Surface *n* (%)	After Caring for a Patient *n* (%)	When Hands Visibly Dirty *n* (%)	After Waste Disposal *n* (%)	After Sneeze/Cough *n* (%)	After Handshake *n* (%)
Water only	165 (41.4)	9 (2.3)	5 (1.3)	31 (7.8)	3 (0.8)	5 (1.3)	18 (4.5)	60 (15.1)	80 (20)
Soap and water	215 (53.9)	365 (91.7)	354 (88.5)	262 (65.7)	213 (53.4)	357 (89.5)	332 (83.2)	102 (25.6)	112 (28)
ABHR	16 (4)	20 (5)	40 (10)	87 (21.8)	175 (43.9)	36 (9)	47 (11.8)	50 (12.6)	107 (26.8)
Disposable handkerchiefs	1 (0.3)	2 (0.5)	1 (0.3)	2 (0.5)	1 (0.3)	1 (0.3)	0 (0)	110 (27.6)	0 (0)
Did not practice	2 (0.5)	2 (0.5)	0 (0)	17 (4.3)	7 (1.8)	0 (0)	2 (0.5)	76 (19.1)	101 (25.3)

ABHR = alcohol-based hand rub.

## References

[1] World Health Organization (2015). Public Health for Mass Gatherings: Key Considerations.

[2] Rajab M.H. (2019). A Master of Public Health with a Concentration in Mass Gatherings Health. Cureus.

[3] World Health Organization WHO Coronavirus Disease (COVID-19) Dashboard. https://covid19.who.int/.

[4] Petersen E., Memish Z.A., Zumla A., Maani A.A. (2020). Transmission of respiratory tract infections at mass gathering events. Curr. Opin. Pulm. Med..

[5] Hoang V.T., Gautret P. (2018). Infectious Diseases and Mass Gatherings. Curr. Infect. Dis. Rep..

[6] Badshah S.L., Ullah A., Badshah S.H., Ahmad I. (2020). Spread of Novel coronavirus by returning pilgrims from Iran to Pakistan. J. Travel Med..

[7] Channel News Asia More Coronavirus Cases in Iran’s Qom as Religious Gatherings Seen at Risk. https://www.channelnewsasia.com/news/world/iran-qom-coronavirus-covid19-religious-gatherings-cases-12455638.

[8] Shim E., Tariq A., Choi W., Lee Y., Chowell G. (2020). Transmission potential and severity of COVID-19 in South Korea. Int. J. Infect. Dis..

[9] Perveen S., Orfali R., Azam M.S.U., Aati H.Y., Bukhari K., Bukhari S.I., Taweel A. (2020). Coronavirus nCOVID-19: A pandemic disease and the Saudi precautions. Saudi Pharm. J..

[10] Ebrahim S.H., Memish Z.A. (2020). Saudi Arabia’s drastic measures to curb the COVID-19 outbreak: Temporary suspension of the Umrah pilgrimage. J. Travel Med..

[11] World Health Organization (WHO) Coronavirus Disease (COVID-19) Advice for the Public. https://www.who.int/emergencies/diseases/novel-coronavirus-2019/advice-for-public.

[12] Balaban V., Stauffer W.M., Hammad A., Afgarshe M., Abd A.M., Ahmed Q., Memish Z.A., Saba J., Harton E., Palumbo G. (2012). Protective practices and respiratory illness among US travelers to the 2009 Hajj. J. Travel Med..

[13] Gautret P., Hai V., Sani S., Doutchi M., Parola P., Brouqui P. (2011). Protective measures against acute respiratory symptoms in French pilgrims participating in the Hajj of 2009. J. Travel Med..

[14] Hashim S., Ayub Z.N., Mohamed Z., Hasan H., Harun A., Ismail N., Rahman Z.A., Suraiya S., Naing N.N., Aziz A.A. (2016). The prevalence and preventive measures of the respiratory illness among Malaysian pilgrims in 2013 Hajj season. J. Travel Med..

[15] Mahdi H., Alqahtani A., Barasheed O., Alemam A., Alhakami M., Gadah I., Alkediwi H., Alzahrani K., Fatani L., Dahlawi L. (2020). Hand Hygiene Knowledge and Practices among Domestic Hajj Pilgrims: Implications for Future Mass Gatherings Amidst COVID-19. Trop. Med. Infect. Dis..

[16] Almofada S.K., Alherbisch R.J., Almuhraj N.A., Almeshary B.N., Alrabiah B., Saffan A., Baseer M.A. (2020). Knowledge, Attitudes, and Practices Toward COVID-19 in a Saudi Arabian Population: A Cross-Sectional Study. Cureus.

[17] Hanawi M.K., Angawi K., Alshareef N., Qattan A.M.N., Helmy H.Z., Abudawood Y., Alqurashi M., Kattan W.M., Kadasah N.A., Chirwa G.C. (2020). Knowledge, Attitude and Practice Toward COVID-19 Among the Public in the Kingdom of Saudi Arabia: A Cross-Sectional Study. Front. Public Health.

[18] Siddiqui A.A., Alshammary F., Amin J., Rathore H.A., Hassan I., Ilyas M., Alam M.K. (2020). Knowledge and practice regarding prevention of COVID-19 among the Saudi Arabian population. Work.

[19] Almutairi A.F., Bani M.A.A., Alessa Y.M., Almutairi S.B., Almaleh Y. (2020). Public trust and compliance with the precautionary measures against COVID-19 employed by authorities in Saudi Arabia. Risk Manag. Healthc. Policy.

[20] World Health Organization Coronavirus Disease (COVID-19) Advice for the Public: Mythbusters. https://www.who.int/emergencies/diseases/novel-coronavirus-2019/advice-for-public/myth-busters#5g.

[21] Centers for Disease Control and Prevention When and How to Wash Your Hands. https://www.cdc.gov/coronavirus/2019-ncov/prevent-getting-sick/prevention.html.

[22] Gharpure R., Hunter C.M., Schnall A.H., Barrett C.E., Kirby A.E., Kunz J., Berling K., Mercante J.W., Murphy J.L., Garcia W.A.G. (2020). Knowledge and Practices Regarding Safe Household Cleaning and Disinfection for COVID-19 Prevention-United States, May 2020. MMWR Morb. Mortal Wkly. Rep..

[23] Mohamed M.S., Moulin T.C., Schiöth H.B. (2020). Sex differences in COVID-19: The role of androgens in disease severity and progression. Endocrine.

[24] Unaib R., Abdullah M.A.S. (2020). Knowledge, Attitude and Practices of Health Care Workers about Corona Virus Disease 2019 in Saudi Arabia. J. Epidemiol. Glob. Health.

[25] Mahmood A., Eqan M., Pervez S., Alghamdi H.A., Tabinda A.B., Yasar A., Brindhadevi K., Pugazhendhi A. (2020). COVID-19 and frequent use of hand sanitizers; human health and environmental hazards by exposure pathways. Sci. Total Environ..

[26] Alqahtani A.S., Tashani M., Heywood A.E., Almohammed A.B.S., Booy R., Wiley K.E., Rashid H. (2020). Tracking Australian Hajj Pilgrims’ Health Behavior before, during and after Hajj, and the Effective Use of Preventive Measures in Reducing Hajj-Related Illness: A Cohort Study. Pharmacy.

[27] Dauda G.M., Hasan H., Naing N.N., Wan A.N., Zeiny D.Z., Nor A.W., Abubakar B.A. (2019). Assessment of Knowledge, Attitude and Practice towards Prevention of Respiratory Tract Infections among Hajj and Umrah Pilgrims from Malaysia in 2018. Int. J. Environ. Res. Public Health.

[28] Alqahtani A.S., Fakeerh M., Bondagji D., Park S., Heywood A.E., Wiley K.E., Booy R., Rashid H. (2018). Hand hygiene compliance and effectiveness against respiratory infections among Hajj pilgrims: A systematic review. Infect. Disord. Drug Targets.

[29] Tobaiqy M., Alhasan A.H., Shams M.M., Amer S.A., Mac L.K., Alcattan M.F., Almudarra S.S. (2020). Assessment of Preventative Measures Practice among Umrah Pilgrims in Saudi Arabia, 1440H-2019. Int. J. Environ. Res. Public Health.

[30] Ng W.K., Shaban R.Z., Mortel T. (2017). Healthcare professionals’ hand hygiene knowledge and beliefs in the United Arab Emirates. J. Infect. Prev..

[31] Beiu C., Mihai M., Popa L., Cima L., Popescu M.N. (2020). Frequent hand washing for COVID-19 prevention can cause hand dermatitis: Management tips. Cureus.

[32] Algaissi A.A., Alharbi N.K., Hassanain M., Hashem A.M. (2020). Preparedness and response to COVID-19 in Saudi Arabia: Building on MERS experience. J. Infect. Public Health.

